# When stress matters most: developmental timing and socio-ecological stressors among Mexican-origin adolescents from low-income immigrant families

**DOI:** 10.1017/S0954579426101539

**Published:** 2026-05-26

**Authors:** Ka I Ip, Wen Wen, Sujin Lee, Lester Sim, Su Yeong Kim

**Affiliations:** 1 Institute of Child Development, University of Minnesota Twin Cities, USA; 2 Crown Family School of Social Work, Policy, and Practice, The University of Chicago, USA; 3 Psychology, University of Michigan, Ann Arbor, USA; 4 School of Social Sciences, Singapore Management University, Singapore; 5 Human Development and Family Sciences, The University of Texas at Austin, USA

**Keywords:** adolescence, developmental timing, immigrant, mental health, stress

## Abstract

This study investigates the dynamic, time-varying associations between multiple socio-ecological stressors and internalizing symptoms among Mexican-origin youth from low-income immigrant families. Grounded in a socioecological framework and employing time-varying effect modeling (TVEM), we examine how stressors at the interpersonal, family, and neighborhood levels differentially influence anxiety and depressive symptoms across early adolescence (ages 11–13), middle adolescence (ages 14–17), and late adolescence/emerging adulthood (ages 18–20). Participants included 604 Mexican-origin adolescents (54% female) from low-income immigrant families, assessed across three waves spanning nine years. Five distinct stressors were identified: discrimination, foreigner stress, economic stress, language brokering stress, and neighborhood violence/non-safety. Results from TVEM analyses revealed that the impact of discrimination on internalizing symptoms was more pronounced during early and middle adolescence, while foreigner stress became increasingly more pronounced in late adolescence/emerging adulthood. Economic hardship and language brokering stress consistently predicted internalizing symptoms across all developmental periods, whereas neighborhood violence/non-safety exerted the greatest influence during early adolescence. These findings underscore the importance of considering how stressor type and developmental timing intersect to shape mental health outcomes. Moreover, the results suggest that identifying sensitive windows for specific socio-ecological stressors can inform the optimal timing of tailored, developmentally sensitive interventions to mitigate their adverse effects.

Mexican immigrant families in the United States often experience disproportionate exposure to chronic stressors across multiple ecological levels – interpersonal, family, and neighborhood) – stemming from their racial–ethnic minority status and immigrant positioning. These socio-ecological stressors, including discrimination, economic hardship and community violence, are embedded in broader structural inequities and can trigger chronic physiological stress responses that can shape developmental trajectories and contribute to mental health disparities (Adam et al., 2020). The present study addresses a critical gap in our understanding of *when* specific types of socio-ecological stressors are most consequential for the emotional well-being of Mexican-origin youth. Specifically, we ask: At what points during adolescence do distinct socio-ecological stressors exert the strongest influence on internalizing symptoms (depression and anxiety) among adolescents from low-income, Mexican immigrant families?

This question is grounded in an emerging body of developmental research that highlights adolescence as a particularly sensitive period for stress exposure (Gee & Casey, 2015). During this period, adolescents undergo rapid neurobiological and psychological reorganization – including neuroendocrine changes, identity formation, increasing autonomy - which heighten their reactivity to environmental inputs (Blakemore, 2012; Dahl et al., 2018; D. G. Gee & Casey, 2015; Umaña-Taylor et al., 2014). Often referred to as a “second window of plasticity,” adolescence is marked by recalibration of stress and emotional systems in response to environmental demands (DePasquale et al., 2021; Gunnar et al., 2019). This highly plastic developmental window coincides with the onset of internalizing problems such as depression and anxiety, underscoring adolescence as a critical period for prevention and intervention efforts (Dahl et al., 2018).

While psychosocial stressors trigger the activation of stress physiological responses designed to ensure organism’s survival (e.g., fight or flight responses), the extent to which certain types of stressors are perceived as potential threats is influenced by both developmental timing (Cohodes et al., 2021; Gee & Ford, 2011) and sociocultural learning (Ip et al., 2021 *).* As such, adolescents’ susceptibility to different forms of socio-ecological stressors may vary based on both their age and the nature of the stressor. For example, racial–ethnic discrimination may have especially deleterious effects on mental health during middle adolescence (∼ages 14–17; Benner et al., 2018), when youth are actively forming racial–ethnic identity (*Umaña-Taylor et al., 2014*) and recognizing how their identity may elicit differential treatment (French et al., 2006; Hughes et al., 2016).

Although recent theoretical models have emphasized developmental timing as a key moderator of stress effects (Cohodes et al., 2021; Gee & Casey, 2015), empirical work has rarely examined how culturally relevant socio-ecological stressors (e.g., discrimination, economic strain, language brokering, or neighborhood disadvantage; Kim, Schwartz, et al., 2018) vary in salience across developmental periods. This is a critical gap in both the research and clinical landscape, as most interventions implicitly treat adolescence as a homogenous period, potentially missing optimal windows for prevention (Cicchetti & Rogosch, 2002). Indeed, insufficient attention to developmental timing may partly explain why mental health interventions for adolescents often yield modest or inconsistent outcomes (Weisz et al., 2017). From a clinical and public health perspective, identifying *when* particular stressors are most consequential is essential for tailoring the timing and content of interventions. Without this knowledge, programs may miss critical developmental opportunities for prevention. Thus, clarifying the developmental timing of different socio-ecological stressors is crucial for designing more effective, targeted strategies to support the mental health of Mexican-origin adolescents from immigrant families and for allocating community resources where they can have the greatest impact.

To address this gap, the present study applies time-varying effect modeling (TVEM; Li et al., 2015) to examine how the associations between chronic socio-ecological stressors and internalizing symptoms unfolds across three developmental periods - early (ages 11–13), middle (14–17), and late adolescence/emerging adulthood (18–20). This approach enables us to identify sensitive windows of vulnerability, or periods when specific stressors exert the greatest influence on internalizing symptoms such as anxiety and depression, thereby providing an empirical basis for more precise and developmentally informed intervention strategies.

Importantly, the developmental salience of stressors likely may vary by cultural, socioeconomic, and immigrant contexts, underscoring the need for examining these processes within a relatively homogeneous group of adolescents who share similar sociocultural and family contexts (Roosa et al., 2008). Accordingly, the present study focuses on Mexican-origin adolescents from low-income immigrant families in Texas. Texas is a state with a large Mexican-origin population and a sociopolitical climate marked by restrictive immigration policies and anti-immigrant sentiment (Noe-Bustamante, 2023; Philbin et al., 2018). Although this study does not assess policy-level factors directly, this broader context likely shapes the occurrence of socio-ecological stressors and how these stressors are interpreted. At the same time, adolescents within this context remain heterogeneous, and personspecific dynamics between stressors and symptoms may diverge from groupaverage patterns; accordingly, the present analyses target grouplevel timing while acknowledging potential individuallevel variability (Fisher et al., 2018; Molenaar & Campbell, 2009; Molenaar, 2004).

In what follows, we first outline an integrative risk and resilience framework that situates the socio-ecological stressors experienced by Mexican-origin adolescents. We then review empirical evidence on the timing of stress exposure and its implications for internalizing symptoms in this population.

## Using an integrative risk and resilience model to understand minority stress exposure among youth

The integrative risk and resilience model provides a developmental framework for understanding how immigrant-origin youths adapt through their interactions with multiple ecological systems (e.g., families, schools, neighborhoods) (Suárez-Orozco et al., 2018). This model underscores that stressors unique to immigrant families, which influence developmental processes, could originate from multiple domains. For Mexican-origin youth, cultural stress often stems from challenges tied to their racial–ethnic identity, including racial/ethnic discrimination (Flores et al., 2010), discrimination because of their skin color (Wang et al., 2022), discrimination in academic settings (Hughes et al., 2016), stereotyping as perpetual foreigners (Armenta et al., 2013; Kim, Schwartz, et al., 2018), and difficulties fitting into mainstream American culture (i.e., cultural misfit; Wen et al., 2022). Additionally, many Mexican-origin adolescents take on language brokering roles, using their bilingual skills to mediate between their parents and US institutions (Kam & Lazarevic, 2014). While this responsibility can foster self-efficacy, it also imposes significant acculturative stress (Kam & Lazarevic, 2014).

Beyond cultural and linguistic challenges, economic hardship disproportionately affects Mexican-origin families, who frequently face lower household incomes compared to the national median (Lopez & Velasco, 2011). This economic disadvantage can stem from structural barriers to employment (e.g., documentation status, limited educational opportunities). Finally, Mexican-origin families are overrepresented in concentrated poverty neighborhoods, where youth are more likely to be exposed to neighborhood violence and feeling unsafe in one’s neighborhood (White et al., 2021).

However, few studies have simultaneously examined the independent roles of multiple stressors across socio-ecological levels, despite evidence that immigrant-origin youth navigate a complex, racially stratified environment . To address this gap, we integrate prominent theoretical frameworks (Coll et al., 1996; Conger et al., 2010; Kim, Schwartz, et al., 2018; Suárez-Orozco et al., 2018; Zou & Cheryan, 2017) to first identify distinct types of stressors at three socioecological levels (interpersonal, family, and neighborhood) that are particularly salient for Mexican-origin adolescents from low-income families.

## Type of stress exposure and developmental racial positionental timing

### Interpersonal level: racial–ethnic discrimination, skin color discrimination, and school discrimination

Mexican-origin youth frequently experience discrimination in both social and institutional settings (Coll et al., 1996; Hughes et al., 2016; Huynh & Fuligni, 2010). Racial discrimination involves unfair treatment based on racial–ethnic background (e.g., being treated with less respect), whereas skin color discrimination reflects biases associated with darker skin tones, reinforcing racialized hierarchies that privilege lighter-skinned individuals. These experiences have been linked to lower self-esteem and distress (Huynh & Fuligni, 2010), altered stress responses (Chen et al., 2024) and increased risk for internalizing symptoms such as depression and anxiety (Benner et al., 2018). In addition, discrimination in schools is particularly harmful, as educational environments play a critical role in shaping sense of belonging, identity, and academic self-efficacy (Berkel et al., 2010; Eccles & Roeser, 2011). Latinx adolescents who experience discrimination in school, such as being assumed to be less intelligent or facing disproportionate disciplinary action, show higher levels of internalizing problems (Benner et al., 2018; Castro et al., 2024).

Mexican-origin youth may be most susceptible to different forms of discrimination (e.g., skin color, academic settings) during early (ages 11–13) and middle (ages 14–17) adolescence. During this period, adolescents begin to develop an awareness of bias, understand social hierarchy and actively explore and form their own racial–ethnic identity and critical consciousness (Mathews et al., 2020). Through family socialization (Hughes et al., 2006) and interaction with peers at school (Byrd & Legette, 2022), youth must reflect upon their identities and visible characteristics (e.g., skin color), which can be a source of discrimination in a racially/ethnically hierarchical and oppressive society. Studies have found that perceived discrimination increases during middle school and tends to decrease during high school, likely reflecting the growing salience of race/ethnicity to adolescents’ identities during this period (Hughes et al., 2016). Supporting this, meta-analytical evidence has shown that discrimination experienced during early and middle adolescence has a stronger effect size than that experienced later in life (Benner et al., 2018).

### Interpersonal level: foreigner stress and cultural misfit

The racial position model (Zou & Cheryan, 2017) further posits that the lived experiences of adolescents from Latinx backgrounds are unique and may not mirror those of other racial/ethnic minoritized groups (e.g., Black and Asian Americans), as they are placed in a social position of being perceived as both inferior and culturally foreign compared to the dominant racial group (i.e., White Americans). **Foreigner stress** occurs when adolescents are perceived as perpetual outsiders, even if they are US-born. These experiences include being questioned about their origins or being assumed to be non-American based on their appearance or accent. Similarly, **cultural misfit** refers to an internalized sense of not belonging due to cultural differences between Mexican-origin youth and mainstream US society (Cozzarelli & Karafa, 1998). While few studies have simultaneously examined both discrimination and perceived foreigner stress/cultural misfit among youth (Kim, Hou, et al., 2018; Wang et al., 2022), research with Latinx adults showed that higher levels of perceived foreigner stress are generally associated with worse mental health outcomes (e.g., elevated depressive symptoms), even after controlling for ethnic discrimination (Armenta et al., 2013). This finding suggests that foreigner stress is conceptually distinct from racial–ethnic discrimination, as it continues to predict mental health beyond the effects of discrimination. In our analysis plan, we therefore include each stressor in TVEMs while controlling for the others, ensuring a conservative test of each stressor’s unique contribution

In contrast to discrimination, it is less clear when youth are more susceptible to the influence of perceived foreigner stress and cultural misfit during adolescent development, as this issue is understudied among youth (Kim, Schwartz, et al., 2018; Wen et al., 2022). Compared to perceived discrimination, perceived foreigner stress and cultural misfit is often more subtle and ambiguous in nature (e.g., “Where are you from?”; Armenta et al., 2013). Therefore, they likely require more advanced social cognition about others’ thoughts and intentions, as well as self-awareness about being recognized as a “foreigner” or internalized feelings of “culturally misfit” in the United States. Adolescents may be most susceptible to the influence of these stressors during late adolescence/emerging adulthood when they can “read between the lines” and recognize others’ intentions and how these intentions relate to their identities as immigrants or children of immigrants. Consequently, we expect that foreigner stress may be linked more strongly to adolescents’ mental health outcomes during late adolescence/emerging adulthood.

### Family level: economic hardship, parental conflict and worry about money

Economic strain is a salient stressor within many Mexican-origin families, manifesting in parental fights about money, difficulties paying bills, and chronic worry about economic security (Mistry et al., 2009). According to the family stress model (Conger et al., 2010), economic stress can disrupt parent–child relationships, increase household tension, and contribute to emotional insecurity among adolescents, heightening their risk for mental health difficulties

Family economic stress may be detrimental to youth mental health through minimizing family and social resources (Conger et al., 2010). We postulate that family economic stress may have a persistent and pervasive effect throughout adolescence due to its chronic, intergenerational, and cascading impact on the entire family (Kavanaugh et al., 2018; Lee et al., 2022)

### Family level: language brokering stress

Language brokering, while often framed as a positive responsibility, can become a stressor when adolescents are required to translate in a variety of places (e.g., school) and situations (e.g., report card) beyond their developmental capacity (Morales & Hanson, 2005; Umaña-taylor, 2003). When youth must translate complex documents for their parents, especially in public settings, they may experience frustration, emotional burden, and role reversal in the parent–child dynamic. These stressors have been linked to heightened anxiety and depressive symptoms (Kim, Hou, et al., 2018)

Children of immigrants typically start language brokering between the ages of 8 and 12 (Morales & Hanson, 2005) and continue to serve as language brokers for their families throughout their development. Stress from language brokering can occur in different places (e.g., supermarket and school) and for different things (e.g., phone calls, report cards). Youth likely possess greater mastery of the English language when they get older, which might buffer against the stress associated with language brokering, suggesting that language brokering stress might be more salient in early adolescence rather than later (Shen et al., 2022). Given that few researchers have investigated the developmental periods during which language brokering stress exacerbates youth mental health problems, coupled with research demonstrating brokering to be a chronic family stressor for youth (Kim, Hou, et al., 2018), we also examined whether there are developmental periods during which youth may be most susceptible to the effect of language brokering stress.

### Neighborhood level: neighborhood violence and non-safety

Mexican-origin youth residing in low-income neighborhoods are disproportionately exposed to community violence (including gang-related incidents) (Mora et al., 2022) and often face frequent police stops and surveillance by law enforcement in their communities (Del Toro et al., 2019). Witnessing or fearing violent and aggressive incidents can result in hypervigilance in adolescents (Miliauskas et al., 2022). Additionally, simply perceiving one’s neighborhood as unsafe, including fear of walking alone or concerns about personal safety, can operate as a chronic stressor that erodes well-being (Robinette et al., 2021). This long-term exposure to unsafe environments may lead to elevated internalizing symptoms, including depression, social withdrawal, and anxiety (Baranyi et al., 2021; White et al., 2021)

Neighborhood violence and neighborhood non-safety may be particularly salient during early adolescence when adolescents begin to have increased autonomy within their neighborhood. At the same time, this is a period when adolescents have decreased reliance of parental protection as a safety net (D. G. Gee, 2021; Sameroff, 2010). Hence, early adolescence may be a developmental period during which adolescents are hypervigilant to the danger in their neighborhood surroundings, increasing their risk for heightened internalizing symptoms.

## The current study

The overall goal of the study is to delineate the influence of the type and developmental timing of stressors on mental health development among Mexican-origin youth. By 2,060, 1 in 3 youth in the United States would be from a Latinx background, with children of Mexican-origin immigrant families representing the largest and fastest growing Hispanic population (Noe-Bustamante et al., 2023). Hence, there is a need to better understand how stressors at multiple socio-ecological levels may influence their mental health development and identify potential “developmental window” during which adolescents may be most susceptible to the influence of specific stressors to optimize the timing for preventive intervention.

Using a confirmatory factor analysis (CFA) approach, our first aim is to group the “type” of stressors that are salient to Mexican-origin youth by examining the factor structures of available contextual stressors, including measures that assess various types of discrimination, foreigner stress, cultural misfit, language brokering stress, economic stress, and neighborhood violence and non-safety. Of note, our goal is not to generate a comprehensive list of stress exposures that Mexican-origin youth may encounter. Rather, we are particularly interested in stressors at different socio-ecological environments (interpersonal, family, and neighborhood) that are theoretically salient to Mexican-origin youth who are racial–ethnic minorities and children of immigrants living in the United States. We hypothesize that we will be able to identify distinct types of stressors operating at different socio-ecological levels.

Second, using TVEMs (Li et al., 2015), we aim to explore the dynamic time-varying and independent associations between identified types of stressors (from Aim 1) and anxiety and depressive symptoms among Mexican-Cultural Estrangement Inventoryorigin youth across early (11–13), middle (14–17), and late (18–20) adolescence and emerging adulthood. TVEM is a nonparametric modeling technique developed for longitudinal repeated measures. TVEM clarifies how the effect of a predictor varies continuously with age/time. While multilevel modeling can be used to study withinperson change, in this study we selected TVEM because the design includes three waves per adolescent and our primary aim was to estimate the stress–internalizing association as a smooth function of age at the group level. Standard multilevel parameterizations would either constrain age effects to linear or loworder forms or require prespecifying spline knots, which are weakly identified with only three time points. By contrast, TVEM estimates the agevarying effect nonparametrically across the full 11–20 age range, allowing us to characterize how these stressors relate to internalizing symptoms (anxiety and depression) without imposing a parametric shape. In other words, this approach captures “developmental windows” across different developmental periods to highlight when youth may be most affected by these stressors (see method below for more details).

At the interpersonal level, we expect that the effect of discrimination on youth internalizing problems may be more pronounced during early (ages 11–13) and middle (ages 14–17) adolescence compared to late adolescence and emerging adulthood (ages 18–20), whereas the effect of foreigner stress may be more pronounced during late adolescence/emerging adulthood. At the family level, we expect that economic stress may have long-lasting effects on adolescents from early to late adolescence, whereas language brokering stress might be more salient in early (rather than later) adolescence. At the neighborhood level, we expect that the effect of neighborhood violence and unsafety may be stronger during early adolescence. We estimate the time-varying effect for each stressor in TVEM while controlling for the others, ensuring a conservative test of each stressor’s independent contribution. We also include gender, nativity, and parental education as covariates in all TVEM models, as these factors have been associated with youth mental health development.

## Method

### Participants

The current study was part of larger three-wave longitudinal research focusing on Mexican-origin immigrant families in the United States. The current study included 604 adolescents who translated for at least one of their parents between Spanish and English (i.e., language brokering) and were from Mexican immigrant families. Among 604 adolescents (*M*_*age*_ = 12.92, *SD* = .92) recruited at Wave 1 from 2012 to 2015, 54% were female and 76% were born in the United States. The average parental highest education level was some middle/junior high school, and the median household income range was from $20,001 and $30,000. Wave 2 included 483 adolescent participants (M_age_ = 13.72, *SD* = .90) and was collected around one year after Wave 1. Wave 3 included 334 adolescent participants (*M*_*age*_ = 17.62, *SD* = 1.05) and was collected around four years after Wave 2. Attrition analysis showed that families in which parents had a higher education level were more likely to remain in the study at Wave 2 (*t*_mother_ (591) = 2.41, *p* < .05; *t*_father_ (291) = 3.13, *p* < .01) and adolescents who were younger at Wave 2 were more likely to remain in the study at Wave 3 (*t*_*age*_ (481) = 2.96, *p* < .01).

### Procedure

The recruitment of participants was initiated in 2012 through school presentations, public records, and community publicity. Research assistants would reach out to potential participants via phone calls to check whether the families were Mexican-origin and had an adolescent child in middle school who translated for their families. Then, research assistants would schedule a visit to families who met the qualification of participation. In the family visit, parents and adolescents would provide informed consent and informed assent, respectively. At each data collection wave, adolescents were asked to answer a set of questions in a family visit. Questions were prepared in both English and Spanish. English questionnaires were translated by bilinguals to Spanish and then back-translated to English. At Wave 1, 2, and 3, families were compensated $60, $90, and $90, respectively.

### Measures

Adolescents self-reported their stress experiences at the individual level (i.e., by racial discrimination, discrimination in academic settings, and discrimination because of skin color, foreigner stress, cultural misfit, and language brokering stress for mothers/fathers in different places/situations), family-level (i.e., parents fighting about money, difficulty paying bills, and worry about money), and neighborhood level (i.e., neighborhood violence and neighborhood non-safety) as well as their internalizing symptoms (i.e., depressive symptoms and anxiety) at all three data collection waves. The mean scores of each scale were calculated to represent the level of each measurement at each wave.

#### Racial discrimination

Racial discrimination was measured by a nine-item scale adapted from the Everyday Discrimination Scale (Williams et al., 1997). Items were modified to focus on youths’ discriminatory experiences specifically related to being of Mexican origin. A sample item included, “I am treated with less courtesy than other people because I am Mexican” (1 = *never*, 4 = *frequently*; α ranges from .88 to .91).

#### Discrimination because of skin color

Discrimination because of skin colors was assessed by one item developed for the current study, “How often are you discriminated because of your skin color?” (1 = *never*, 5 = *always*) (Wang et al., 2022).

#### Discrimination in academic settings

Discrimination in academic settings was measured by three items assessing academic difficulties related to minority ethnicity-related discrimination (Fisher et al., 2000; McNeilly et al., 1996). One item was adopted from the Educational Discrimination Distress Subscale (Fisher et al., 2000): “I was wrongly disciplined or given after-school detention.” The other two items were adapted from discrimination in the academic domain of the Perceived Racism Scale (McNeilly et al., 1996): “Teachers and students assume I’m less intelligent,” and “My academic advancement has suffered because I am Mexican” (1 = *strongly disagree*, 5 = *strongly agree*; α ranges from .59 to .64).

#### Foreigner stress

Foreigner stress was assessed by a four-item scale developed by Kim et al. (2018). Sample items included, “Because of how I speak, people sometimes assume I am not a U.S. American” (1 = *strongly disagree*, 5 = *strongly agree*; α ranges from .71 to .78).

#### Cultural misfit

Cultural misfit was measured by a four-item subscale from the Cultural Estrangement Inventory (Cozzarelli & Karafa, 1998). Sample items included, “I feel that somehow I don’t fit in with U.S. Americans”(1 = *strongly disagree*, 5 = *strongly agree*; α is .77 for each wave, respectively).

#### Language brokering stress

Language brokering stress for mothers/fathers in different places was measured by seven items (Zhang et al., 2020). Adolescents were asked, “How stressful is it to translate from English to Spanish for your mother/father at the following places?”. Sample places included, “at home,” “At your school/at parent and teacher conferences,” and “at stores (supermarket) “ (1 = *not stressful*, 5 = *extremely stressful*; α ranges from .89 to .93 for brokering for mothers, and .89 to .92 for brokering for fathers). Answers were coded as missing if adolescents self-reported they did not translate for mothers/fathers in the specified places.

Language brokering stress for mothers/fathers in different situations was measured by eleven items, respectively (Shen et al., 2019). Adolescents were asked, “How stressful is it to translate from English to Spanish the following things for your mother/father?”. Sample situations included, “your homework,” “Report cards or school progress reports/ other school information,” and “phone calls” (1 = *not stressful*, 5 = *extremely stressful*; α ranges from .93 to .95 for brokering for mothers, and .93 to .94 for brokering for fathers). Answers were coded as missing if adolescents self-reported they didn’t translate for mothers/fathers in the specified situations.

#### Parents fighting about money

Parents fighting about money was measured by three items from a previous study (Mistry et al., 2009). Sample items included, “Did your parents argue with each other about not having enough money?” (1 = *never*, 5 = *always*; α ranges from .71 to .83).

#### Difficulty paying bills

Difficulty paying bills was measured by one item adopted from a previous study (Mistry et al., 2009), which was, “How much of a problem did your family have because your parents did not have enough money to buy things your family needs or wants?” (1 = *not at all*, 5 = *very*).

#### Worry about money

Worry about money was measured by one item adopted from a previous study (Mistry et al., 2009), which was, “How upset or worried were your parent(s) because they did not have enough money to pay for things?” (1 = *not at all*, 5 = *very*).

#### Neighborhood violence

Neighborhood Violence was measured by three items adopted from Survey of Children’s Exposure to Community Violence (Richters & Saltzman, 1990). Adolescents were asked about their perceptions on whether people in their neighborhood did the following things. Sample items were, “Get into physical fights (slap, punch, or hit each other),” “Are picked up, arrested, or taken away by the police,” and “Offer, sell, buy or use illegal drugs” (1 = *never*, 5 = *always*; α ranges from .71 to .83).

#### Neighborhood unsafety

Neighborhood unsafety was measured by four items adopted from a previous study (Kim et al., 2009). Items were, “My neighborhood is safe for children during the daytime (reverse-coded),” “My neighborhood is safe for children during the nighttime (reverse-coded),” “It is safe in my neighborhood (reverse-coded),” and “I do not feel safe when walking to the school, park, or store in this neighborhood” (1 = *strongly disagree*, 5 = *strongly agree*; α ranges from .73 to .78).

#### Depressive symptoms

Depressive symptoms were measured by the 20-item CES-D scale (Radloff, 1977). Sample items included, “I thought my life had been a failure” and “I did not feel like eating; my appetite was poor” (0 = rarely or none, 3 = most or all; α ranges from .83 to .87).

#### Anxiety

Anxiety was measured by a four-item scale from prior studies (Reynolds & Richmond, 1978; Spitzer et al., 2006). Adolescents were asked, “Over the last 2 weeks, how often have you been bothered by the following problems?”. Items were “feeling nervous,” “having trouble relaxing,” “worrying about what is going to happen,” and “becoming easily annoyed or irritable” (0 = *mot at all,* 3 = *nearly every day*; α ranges from .75 to .81).

#### Covariates

Adolescent self-reported nativity and gender, as well as parent-reported highest education level, were included as covariates in the current study.

### Analytical plan

We first report descriptive statistics and intercorrelation of all study variables. Aim 1. To group distinct type of stressors among Mexican American youth, CFA was conducted in Mplus 8.0 based on the hypotheses. At each wave, stress-related variables were included in the CFA model to construct five latent stress factors – discrimination, foreigner stress, economic stress, language brokering stress, and neighborhood unsafety. Models were evaluated based on the model fit indices recommended by Marsh et al. (1996). For models with good model fit indices (i.e., CFI and TLI are above .9, RMSEA is less than .08, SRMR is less than .05, and factor loading of each variable is higher than .4), factor scores were extracted to represent the latent stress factors.

Aim 2. To investigate the *timing* in which stress is the most influential on adolescents’ internalizing symptoms, we applied TVEMs to examine the association between the stress variables and individual internalizing symptoms across the full age range of the current sample across the three waves (from 11.08 to 21.22 years old).

The stressor variables included in the TVEM analyses were the latent factors (i.e., extracted factor scores) derived from CFA, rather than individual manifest items, allowing for reduced measurement error and more accurate representation of the underlying constructs. Each record in the analytic data set represents one observation for one youth at their observed age. Youth contributed between one and three observations across waves, resulting in an unbalanced longitudinal structure with uneven age coverage across participants. TVEMs are well suited for such data and do not require the same individuals to be observed at every age point. Instead, TVEM estimates age-varying associations by pooling information across participants and waves, leveraging age-indexed variation in the data. Developmental inferences are based on the assumption that youth observed at different ages are drawn from the same underlying population, as in accelerated longitudinal and cohort-sequential designs; accordingly, age-specific estimates reflect a combination of within-person and between-person information.

Age-varying effects were modeled using penalized spline (P-spline) smoothing, which borrows strength across adjacent ages to produce stable and continuous estimates even when sample composition varies across ages. Robust standard errors, analogous to those used in generalized estimating equations, were applied to account for clustering of repeated observations within individuals. A P-spline with 10 knots was used to balance model parsimony and flexibility in capturing time-varying effects (Lanza et al., 2010). Because only three adolescents were older than age 20, results are interpreted within the observed age range of 11.08 to 20.00 years. Accordingly, the age axis reflects pooled observations across all waves, with smoothing and robust variance estimation ensuring valid developmental inferences despite uneven age coverage.

TVEM is intended for nonparametric estimates of continuous change of intercepts or slopes over time after accounting for the nonindependence of data given the nested data structure (i.e., repeated measure within participants). Specifically, we used the %TVEM program version 3.1.1 based on SAS language (Li et al., 2015) to estimate the following model:

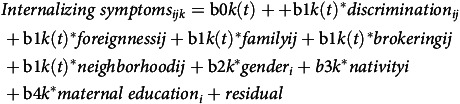




The adjustment of participant *i* at age *j* for outcome *k* (i.e., anxiety or depressive symptoms) was predicted by individual time-varying stress (e.g., discrimination or family stress) at age j while controlling for the time-invariant effect of demographic information of participant i (e.g., nativity) as well as other stressors in the model to conservatively test for the unique effect of each stressor. A total of two TVEM models were tested for anxiety and depressive symptoms, separately. TVEM estimates the unstandardized regression coefficients of the time-varying effects (i.e., *b*s) and the 95% confidence interval (CI) for each *b* at any given age *t*. A time-varying effect at a given time point is considered significant if the CI at that time point does not include zero. For example, the time-varying effect of stress (*b*_*1k*_) represents the association between discrimination and internalizing symptoms (e.g., anxiety) at a given time point (e.g., *t* = 12). If the CI of *b*_*1k*_ is above zero at *t* = 12, there is a significant positive association between discrimination and anxiety at age 12. A rising spline means the association strength increases relative to its own earlier levels. We focused on the descriptive trends of the stress-internalizing associations across time. It is important to note that, in this specification, the TVEMs estimate smooth sample-level functions of time (with confidence bands reflecting estimation uncertainty) and do not separate within- from between-person components, nor recover person-specific trajectories.

## Results

### Descriptive information and correlation

Table 1 shows the descriptive information and correlation among all study variables across three waves. Overall, different stress variables were positively related to each other within the same wave, and the same stress variables were positively related to each other across different waves. Higher levels of stress were significantly associated with more depressive symptoms and higher anxiety at the same wave. In addition, being born in the United States. was associated with less anxiety and depressive symptoms at Wave 2 compared to being born in Mexico; and being male was related to less depressive symptoms and anxiety across three waves compared to being female, except for Wave 3 depressive symptoms.

**Table 1.** Descriptive information and correlation of study variables

	1	2	3	4	5	6	7	8	9	10	11	12	M	SD	Skewness	Kurtosis
1. Nativity (0 = Mexico)	–											–	–	–	–	–
2. Sex (0 = Female)	−.02	–											–	–	–	–
3. Parental education	−.04	.01	–										0.00	2.04	0.47	−0.20
4. W1 racial discrimination	.00	.09*	.02	–									1.37	0.47	1.68	2.66
5. W1 discrimination because of skin color	.02	.06	.00	.46**	–								1.46	0.74	1.71	2.59
6. W1 discrimination in academic settings	−.05	.15**	−.04	.38**	.27**	–							1.84	0.65	0.47	0.02
7. W1 Cultural Misfit	−.08*	.07	−.03	.42**	.27**	.38**	–						2.31	0.67	0.24	0.20
8. W1 Foreigner Stress	−.09*	.06	−.08	.34**	.29**	.35**	.58**	–					2.43	0.73	0.14	0.04
9. W1 Parents fighting about money	.03	−.01	.03	.21**	.21**	.24**	.16**	.19**	–				1.66	0.69	1.17	1.09
10. W1 Difficulty paying bills	.07	.05	.03	.18**	.14**	.19**	.20**	.16**	.46**	–			2.00	0.94	0.92	0.72
11. W1 Worry about money	.06	−.04	.02	.17**	.15**	.10*	.14**	.09*	.36**	.54**	–		2.37	1.14	0.68	−0.26
12. W1 LB Stress for Mother (different places)	−.04	−.12**	−.05	.15**	.07	.00	.13**	.12**	.20**	.17**	.14**	–	1.96	0.78	0.85	0.46
13. W1 LB Stress for Mother (different materials)	−.04	−.10*	−.03	.18**	.12*	.05	.15**	.14**	.21**	.22**	.21**	.74**	1.83	0.73	1.12	1.38
14. W1 LB Stress for Father (different places)	−.03	−.05	−.06	.25**	.15**	.10*	.19**	.16**	.26**	.22**	.23**	.62**	1.68	0.80	1.53	2.31
15. W1 LB Stress for Father (different materials)	−.07	−.04	−.04	.25**	.16**	.07	.20**	.18**	.23**	.24**	.22**	.59**	1.70	0.76	1.59	2.88
16. W1 Neighborhood violence	.03	.05	−.05	.27**	.17**	.32**	.23**	.23**	.20**	.13**	.15**	.03	2.07	0.91	0.81	0.39
17. W1 Neighborhood unsafety	.06	−.05	.02	.23**	.15**	.24**	.21**	.13**	.17**	.12**	.17**	.11*	2.45	0.70	0.05	−0.10
18. W2 Racial discrimination	−.04	.02	.07	.54**	.30**	.29**	.31**	.24**	.13**	.09*	.03	.10*	1.37	0.47	1.56	2.06
19. W2 Discrimination because of skin color	.05	.06	−.04	.34**	.38**	.24**	.19**	.18**	.17**	.11*	.04	.01	1.44	0.69	1.78	3.86
20. W2 discrimination in academic settings	.02	.12*	−.08	.26**	.15**	.44**	.25**	.26**	.10*	.09*	.01	.06	1.81	0.61	0.33	−0.38
21. W2 Cultural Misfit	−.02	−.01	−.04	.29**	.16**	.25**	.42**	.36**	.04	.11*	.11*	.06	2.24	0.65	0.17	0.07
22. W2 Foreigner Stress	−.07	.03	−.05	.25**	.19**	.24**	.36**	.51**	.11*	.08	−.02	.14*	2.42	0.73	−0.04	−0.44
23. W2 Parents fighting about money	.00	−.08	.08	.16**	.16**	.13*	.15**	.18**	.46**	.32**	.22**	.11*	1.65	0.68	1.12	0.90
24. W2 Difficulty paying bills	.00	−.03	.01	.18**	.15**	.22**	.22**	.21**	.36**	.35**	.33**	.17**	1.79	0.85	1.19	1.80
25. W2 Worry about money	−.01	−.17**	.02	.16**	.08	.10*	.14**	.14**	.31**	.32**	.34**	.21**	2.09	0.97	0.72	0.20
26. W2 LB Stress for Mother (different places)	−.12**	−.16**	−.08	.14**	.07	.14**	.20**	.25**	.17**	.10**	.10**	.46**	1.82	0.76	0.85	0.29
27. W2 LB Stress for Mother (different materials)	−.15**	−.15**	−.07	.21**	.12*	.14**	.23**	.27**	.18**	.15**	.14*	.39**	1.75	0.70	1.04	0.76
28. W2 LB Stress for Father (different places)	−.15**	−.05	.00	.25**	.10	.16**	.25**	.23**	.20**	.17**	.16**	.39**	1.60	0.73	1.45	2.09
29. W2 LB Stress for Father (different materials)	−.10**	−.08	−.02	.23**	.15**	.21**	.19**	.19**	.25**	.18**	.13**	.38**	1.62	0.67	1.22	1.38
30. W2 Neighborhood violence	.00	.00	−.03	.16**	.09	.32**	.15**	.18**	.10*	.03	.02	−.03	2.11	0.93	0.70	0.02
31. W2 Neighborhood unsafety	.08	−.02	−.01	.13**	.10*	.21**	.12*	.06	.14**	.06	.08	.08	2.39	0.66	0.16	0.09
32. W3 Racial discrimination	−.13**	−.03	.12*	.27**	.21**	.11	.18**	.21**	.19**	.07	.11*	.02	1.43	0.52	1.54	2.98
33. W3 Discrimination because of skin color	−.11**	.04	.04	.10	.18**	−.04	.14**	.15**	.09	.02	.05	−.02	1.52	0.73	1.44	2.33
34. W3 discrimination in academic settings	−.01	.05	.02	.11*	.10	.10	.13**	.20**	.05	−.01	.00	.10	1.82	0.60	0.53	0.46
35. W3 Cultural Misfit	−.09	.01	.03	.23**	.19**	.11	.28**	.25**	.08	.10	.12*	.03	2.42	0.66	0.52	0.49
36. W3 Foreigner Stress	−.08	.03	−.02	.11	.13*	.08	.17**	.35**	.12*	−.03	.00	.05	2.45	0.74	0.36	0.17
37. W3 Parents fighting about money	−.02	−.12*	.09	.16**	.17**	.04	.09	.15**	.29**	.18**	.19**	.08	1.75	0.74	0.97	0.30
38. W3 Difficulty paying bills	−.04	−.01	.05	.17**	.14*	.07	.19**	.21**	.20**	.29**	.28**	.13*	1.74	0.86	1.07	0.80
39. W3 Worry about money	−.02	−.13*	.07	.23**	.18**	.07	.14**	.16**	.16**	.27**	.26**	.09	1.96	1.10	1.09	0.40
40. W3 LB Stress for Mother (different places)	−.14**	−.18**	−.02	.13*	.14*	−.03	.17**	.18**	.11*	.11*	.03	.31**	1.88	0.71	0.76	0.06
41. W3 LB Stress for Mother (different materials)	−.10	−.16**	.04	.06	.07	−.02	.07	.09	.14*	.07	.04	.24**	1.65	0.58	0.92	0.19
42. W3 LB Stress for Father (different places)	−.02	−.09	.04	.04	.08	−.03	−.02	.05	.21**	.06	.09	.28**	1.63	0.67	1.09	0.60
43. W3 LB Stress for Father (different materials)	−.01	−.10	.09	.10	.12	.00	.08	.11	.17**	.04	.05	.23**	1.63	0.62	1.25	1.80
44. W3 Neighborhood violence	−.02	.10	.00	.10	−.01	.08	.07	.09	.08	.03	.03	.11	2.01	0.90	0.76	0.10
45. W3 Neighborhood unsafety	.11	−.06	.01	.05	−.01	.06	.07	.00	.00	.10	.10	.07	2.30	0.65	0.41	0.94
46. W1 Depressive symptoms	−.07	−.08*	.05	.33**	.17**	.24**	.28**	.20**	.34**	.27**	.25**	.29**	1.56	0.38	1.50	3.01
47. W2 Depressive symptoms	−.09*	−.19**	.06	.20**	.08	.17**	.19**	.13**	.14**	.09	.13*	.14*	1.55	0.39	1.50	2.85
48. W3 Depressive symptoms	−.01	−.08	.08	.23**	.20**	.03	.20**	.15**	.05	.06	.05	.04	1.55	0.38	1.92	6.11
49. W1 Anxiety	−.02	−.10*	.07	.35**	.21**	.15**	.28**	.22**	.34**	.24**	.31**	.29**	1.69	0.61	1.05	1.01
50. W2 Anxiety	−.11*	−.19**	.09	.24**	.13**	.09*	.15**	.14**	.17**	.10*	.15**	.20**	1.72	0.65	0.92	0.28
51. W3 Anxiety	−.06	−.17**	.08	.21**	.19**	.04	.19**	.14**	.14**	.08	.13*	.10	1.85	0.67	0.71	0.02

	13	14	15	16	17	18	19	20	21	22	23	24	25	26	27	28	29	30	31	32
13. W1 LB Stress for Mother (different materials)	–																			
14. W1 LB Stress for Father (different places)	.69**	–																		
15. W1 LB Stress for Father (different materials)	.75**	.75**	–																	
16. W1 Neighborhood violence	.06	.10*	.12**	–																
17. W1 Neighborhood unsafety	.08	.15**	.14**	.45**	–															
18. W2 Racial discrimination	.09	.12**	.14**	.22**	.15**	–														
19. W2 Discrimination because of skin color	.00	.09	.02	.17**	.15**	.47**	–													
20. W2 discrimination in academic settings	.04	.05	.02	.25**	.14**	.34**	.25**	–												
21. W2 Cultural Misfit	.09	.06	.15**	.17**	.15**	.37**	.24**	.39**	–											
22. W2 Foreigner Stress	.12*	.09	.15**	.07	.06	.30**	.21**	.37**	.57**	–										
23. W2 Parents fighting about money	.12**	.17**	.12**	.18**	.12**	.27**	.20**	.20**	.16**	.16**	–									
24. W2 Difficulty paying bills	.23**	.24**	.22**	.20**	.17**	.22**	.15**	.12**	.21**	.17**	.53**	–								
25. W2 Worry about money	.21**	.23**	.16**	.15**	.13**	.16**	.11*	.09	.18**	.14**	.47**	.63**	–							
26. W2 LB Stress for Mother (different places)	.48**	.41**	.43**	.12**	.10*	.16**	.03	.11*	.17**	.18**	.21**	.21**	.29**	–						
27. W2 LB Stress for Mother (different materials)	.48**	.45**	.50**	.15**	.11**	.17**	.10*	.11*	.19**	.19**	.23**	.25**	.29**	.75**	–					
28. W2 LB Stress for Father (different places)	.42**	.52**	.51**	.13**	.18**	.19**	.12*	.04	.16**	.13*	.21**	.27**	.27**	.62**	.69**	–				
29. W2 LB Stress for Father (different materials)	.45**	.43**	.52**	.17**	.16**	.29**	.16**	.13*	.18**	.17**	.27**	.34**	.33**	.60**	.73**	.74**	–			
30. W2 Neighborhood violence	.00	.01	.05	.59**	.28**	.25**	.13**	.38**	.28**	.19**	.17**	.13**	.09	.13**	.15**	.07	.18**	–		
31. W2 Neighborhood unsafety	.10*	.09	.06	.31**	.50**	.15**	.18**	.24**	.19**	.11*	.14**	.17**	.07	.11*	.06	.12*	.18**	.42**	–	
32. W3 Racial discrimination	.06	.06	.01	.07	.04	.38**	.24**	.17**	.18**	.28**	.25**	.25**	.21**	.00	.10	.13*	.10	.08	.05	–
33. W3 Discrimination because of skin color	.02	−.04	−.03	−.03	−.02	.13*	.11*	−.03	.07	.10	.08	.00	−.02	.00	.06	−.04	−.03	.00	−.07	.48**
34. W3 discrimination in academic settings	.09	.05	.05	.10	.06	.24**	.11*	.32**	.24**	.32**	.16**	.21**	.06	.00	.05	.08	.08	.22**	.13*	.39**
35. W3 Cultural Misfit	.02	.07	.00	.11*	.13*	.31**	.19**	.09	.32**	.27**	.07	.15**	.14**	.04	.11	.17**	.11	.14*	.04	.43**
36. W3 Foreigner Stress	.07	.01	.01	.06	.06	.14*	.09	.14**	.18**	.40**	.02	.10	.05	.03	.12*	.06	.04	.07	−.02	.37**
37. W3 Parents fighting about money	.09	.07	.08	.14*	.12*	.21**	.06	.05	.11	.10	.34**	.24**	.30**	.08	.08	.06	.09	.12*	.09	.24**
38. W3 Difficulty paying bills	.11	.15*	.16**	.14*	.13*	.17**	.05	.04	.14*	.17**	.37**	.45**	.44**	.11	.18**	.21**	.19**	.09	.04	.31**
39. W3 Worry about money	.12*	.14**	.11	.05	.18**	.15**	.06	.05	.10	.13*	.36**	.43**	.42**	.08	.15**	.10	.12	.02	.05	.31**
40. W3 LB Stress for Mother (different places)	.25**	.26**	.19**	.02	.08	.14*	.08	.07	.03	.15**	.14*	.18**	.26**	.41**	.41**	.34**	.30**	.01	.02	.20**
41. W3 LB Stress for Mother (different materials)	.24**	.27**	.26**	.05	.13*	.14*	.07	.01	.00	.06	.18**	.21**	.21**	.29**	.43**	.33**	.40**	.02	.01	.21**
42. W3 LB Stress for Father (different places)	.24**	.24**	.25**	.03	−.04	.09	.02	−.02	−.10	−.01	.10	.16**	.19**	.32**	.25**	.29**	.31**	.03	−.05	.20**
43. W3 LB Stress for Father (different materials)	.23**	.26**	.27**	.04	−.02	.15*	.01	−.02	.00	.06	.09	.20**	.20**	.26**	.24**	.33**	.41**	.02	−.05	.21**
44. W3 Neighborhood violence	.07	.04	.06	.36**	.12*	.13*	.09	.18**	.14*	.16**	.00	.09	−.01	.03	.06	.06	.12	.37**	.23**	.13*
45. W3 Neighborhood unsafety	.05	−.01	.04	.14*	.24**	.14*	.13*	.10	.15**	.08	.04	.16**	.09	.05	.06	.09	.12	.12*	.39**	.14*
46. W1 Depressive symptoms	.32**	.37**	.33**	.18**	.28**	.24**	.19**	.20**	.21**	.16**	.20**	.25**	.26**	.24**	.26**	.33**	.31**	.14*	.24**	.11*
47. W2 Depressive symptoms	.19**	.19**	.18**	.17**	.15**	.25**	.17**	.20**	.23**	.17**	.29**	.25**	.25**	.22**	.22**	.25**	.23**	.25**	.23**	.18**
48. W3 Depressive symptoms	.06	.08	.06	.07	.22**	.21**	.12*	.07	.22**	.25**	.18**	.18**	.20**	.03	.11*	.13*	.13*	.14*	.17**	.36**
49. W1 Anxiety	.33**	.36**	.29**	.19**	.27**	.20**	.19**	.14*	.17**	.13*	.22**	.26**	.26**	.28**	.26**	.32**	.22**	.12*	.15**	.15**
50. W2 Anxiety	.24**	.21**	.20**	.09	.12*	.24**	.20**	.12*	.20**	.15**	.31**	.24**	.31**	.30**	.31**	.33**	.31**	.18**	.15**	.23**
51. W3 Anxiety	.15**	.15*	.12*	.11*	.17**	.25**	.09	.07	.19**	.21**	.18**	.22**	.25**	.14*	.19**	.22**	.22**	.18**	.15*	.33**

	33	34	35	36	37	38	39	40	41	42	43	44	45	46	47	48	49	50	51
33. W3 Discrimination because of skin color	–																		
34. W3 discrimination in academic settings	.21**	–																	
35. W3 Cultural Misfit	.28**	.37**	–																
36. W3 Foreigner Stress	.23**	.36**	.41**	–															
37. W3 Parents fighting about money	.10	.21**	.20**	.10	–														
38. W3 Difficulty paying bills	.19**	.23**	.25**	.20**	.53**	–													
39. W3 Worry about money	.16**	.19**	.24**	.12*	.57**	.73**	–												
40. W3 LB Stress for Mother (different places)	.11*	.06	.19**	.18**	.23**	.29**	.25**	–											
41. W3 LB Stress for Mother (different materials)	.02	.06	.12*	.16**	.22**	.24**	.22**	.68**	–										
42. W3 LB Stress for Father (different places)	.07	.07	.10	.07	.20**	.20**	.13*	.59**	.61**	–									
43. W3 LB Stress for Father (different materials)	.10	.11	.18**	.14*	.20**	.19**	.19**	.50**	.64**	.71**	–								
44. W3 Neighborhood violence	.10	.20**	.15**	.15**	.09	.09	.03	.05	.05	.12	.08	–							
45. W3 Neighborhood unsafety	.03	.22**	.18**	.09	.10	.13*	.12*	.08	.03	.03	−.03	.45**	–						
46. W1 Depressive symptoms	.02	.03	.18**	.07	.18**	.16**	.19**	.26**	.19**	.09	.15*	.14*	.25**	–					
47. W2 Depressive symptoms	.03	.08	.15**	.13*	.21**	.16**	.17**	.18**	.16**	.03	.15*	.15**	.20**	.53**	–				
48. W3 Depressive symptoms	.21**	.21**	.38**	.24**	.24**	.29**	.34**	.25**	.23**	.19**	.27**	.17*	.26**	.27**	.35**	–			
49. W1 Anxiety	.05	.07	.17**	.11	.23**	.13*	.16**	.23**	.20**	.18**	.15*	.13*	.17**	.61**	.40**	.30**	–		
50. W2 Anxiety	.08	.13*	.21**	.17**	.22**	.19**	.13*	.17**	.21**	.15*	.15*	.11*	.12*	.41**	.63**	.30**	.51**	–	
51. W3 Anxiety	.23**	.18**	.38**	.17**	.36**	.31**	.36**	.30**	.28**	.27**	.31**	.14*	.15**	.25**	.33**	.68**	.32**	.39**	–

Note. LB = Language Brokering, W1 = Wave 1; W2 = Wave 2; W3 = Wave 3. *p < .05; **p < .01; ***p < .001.

## Aim 1. Confirmatory factor analysis (CFA) of stressors

**Figure 1.**
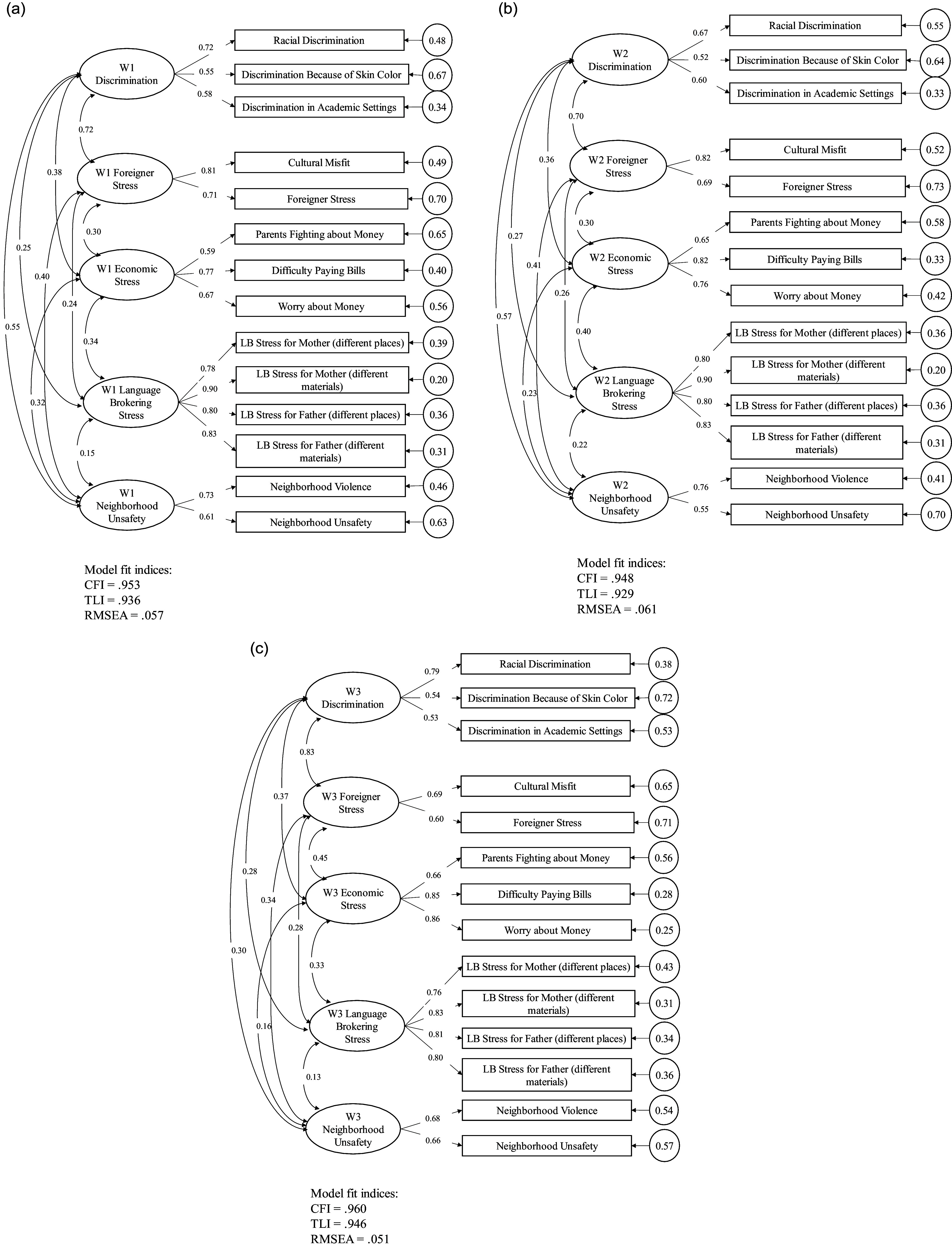
Confirmative factor analysis results of stress variables at each wave. *Note.* W = wave; LB = language brokering.

Using CFA, we found a five-factor latent construct model (we labeled them as discrimination, foreigner stress, economic stress, language brokering stress and neighborhood violence/non-safety) consistently demonstrated a good model fit across 3 waves. Figure 1 depicts the standardized results of three five-factor CFA models of stress variables for all three waves. CFI and TFI for each model were above .90, RMSEA for each model was less than .07, and factor loadings of variables were all above .50. Factor loadings of each item were all above .50.

## Aim 2. Time-varying effects of stressors on adolescent internalizing symptoms

**Figure 2.**
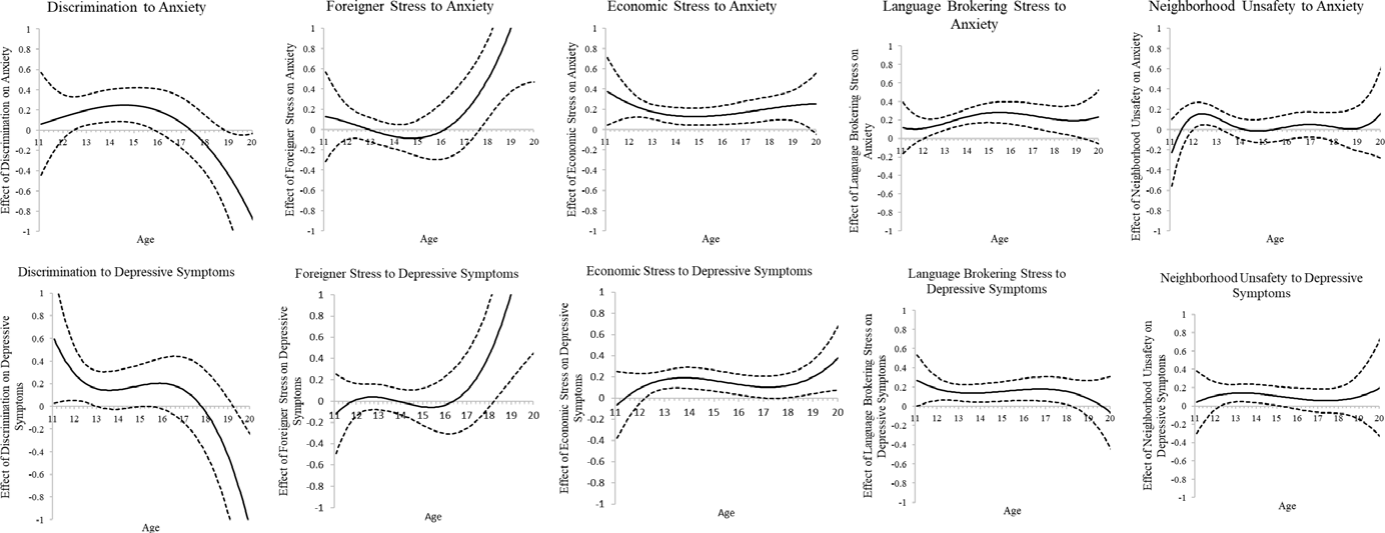
*Time-varying associations between each stressor and adolescents’ anxiety or depressive symptoms*. *Note*. The x-axis represents adolescent age, and the y-axis represents the standardized coefficient of the association between each stressor and internalizing symptoms. The solid line depicts the estimated standardized unique effect of each stressor on anxiety or depressive symptoms across age, adjusted for nativity, gender, parental education and the shared variance among all stressors. The dotted lines indicate the 95% confidence interval (CI) of the estimated effects. Positive associations are observed when both dotted lines (i.e., 95% CI) remain above the x-axis, and negative associations when both are below the x-axis. The total variance explained by all stressors was *R*^2^ = 0.2329 for the anxiety model and *R*^2^ = 0.2270 for the depressive symptoms model.

Figure 2 displays how the strength of the association between each stressor (identified from Aim 1) and adolescent anxiety or depressive symptoms (i.e., *y*-axis values; standardized coefficients) varied across developmental ages, after adjusting for covariates including nativity, gender, and parental education, as well as the shared variance among other stressors in the model. Overall, stressors were positively associated with adolescents’ internalizing symptoms, and the magnitude of these associations changed with age. Together, the time-varying stressors accounted for 23.29% of the variance in anxiety and 22.70% of the variance in depressive symptoms, independent of the effects of covariates (see Figure 2).

### Interpersonal level

Discrimination was related to higher anxiety during early and middle adolescence, particularly between ages 12.52 (*B* = 0.17, 95% CI [0.01, 0.33]) and 15.80 (*B* = 0.21, 95% CI [0.01, 0.41]). However, discrimination was also related to *lower* anxiety in late adolescence, particularly between ages 18.87 (*B* = −0.39, 95% CI [−0.79, −0.00]) and 20.00 (*B* = −0.86, 95% CI [−1.69, −0.03]). In terms of depressive symptoms, discrimination was associated with more depressive symptoms during early adolescence, particularly between ages 11.08 (*B* = 0.59, 95% CI [0.03, 1.15]) and 12.93 (*B* = 0.16, 95% CI [0.01, 0.32]). Conversely, discrimination was linked to *lower* depressive symptoms during late adolescence, particularly between ages 19.28 (*B* = −0.58, 95% CI [−1.15, −0.01]) and 20.00 (*B* = −1.05, 95% CI [−1.86, −0.24]).

Foreigner stress was related to higher anxiety during late adolescence, particularly between ages 17.74 (*B* = 0.38, 95% CI [0.02, 0.75]) and 20.00 (*B* = 1.71, 95% CI [0.47, 2.96]). Additionally, foreigner stress was associated with more depressive symptoms during late adolescence, particularly between ages 18.25 (*B* = 0.55, 95% CI [0.01, 1.09]) and 20.00 (*B* = 1.90, 95% CI [0.45, 3.34]).

### Family level

Economic stress was related to higher anxiety throughout early to late adolescence, particularly between ages 11.08 (*B* = 0.37, 95% CI [0.04, 0.71]) and 19.69 (*B* = 0.25, 95% CI [0.02, 0.49]). It was also associated with higher levels of depressive symptoms throughout early to middle adolescence, particularly between ages 12.21 (*B* = 0.12, 95% CI [0.00, 0.23]) and 17.02 (*B* = 0.10, 95% CI [0.00, 0.21]).

Similarly, language brokering stress was related to higher anxiety throughout early to late adolescence, particularly between ages 12.11 (*B* = 0.11, 95% CI [0.01, 0.21]) and 19.28 (*B* = 0.19, 95% CI [0, 0.39]). Language brokering stress was also associated with higher depressive symptoms throughout early to late adolescence, particularly between ages 11.08 (*B* = 0.27, 95% CI [0.00, 0.54]) and 18.15 (*B* = 0.15, 95% CI [0.01, 0.28]).

### Neighborhood level

Neighborhood violence/non-safety was associated with higher anxiety during early adolescence, particularly between ages 12.01 (*B* = 0.13, 95% CI [0.00, 0.26]) and 13.13 (*B* = 0.10, 95% CI [0.01, 0.19]). It was also linked to more depressive symptoms during early to middle adolescence, particularly between ages 12.31 (*B* = 0.12, 95% CI [0.01, 0.24]) and 15.08 (*B* = 0.11, 95% CI [0.00, 0.21]).

## Discussion

The overarching goal of our study was to disentangle the dynamic, time-varying associations between socio-ecological stressors and internalizing symptoms among Mexican-origin youth from low-income immigrant families. Grounded in key theoretical frameworks (Coll et al., 1996; Conger et al., 2010; Kim, Schwartz, et al., 2018; Sameroff, 2010; Suárez-Orozco et al., 2018; Zou & Cheryan, 2017), we identified five distinct stressors: discrimination, foreigner stress, economic hardship, language brokering stress, and neighborhood violence/unsafety, that operate across interpersonal, familial, and neighborhood levels. Using TVEM, we revealed that the mental health consequences of these stressors shift developmentally: discrimination peaks in early and middle adolescence, foreigner stress intensifies in late adolescence/emerging adulthood, economic, and language brokering stress exert persistent effects throughout adolescence, and neighborhood violence/unsafety is most detrimental in early adolescence. These findings underscore the need for developmentally tailored and contextually sensitive interventions that address the unique challenges Mexican-origin youth face at different periods of their maturation, while also advocating for systemic changes to reduce structural inequities.

Consistent with our first hypothesis, we identified distinct types of stressors salient to Mexican-origin youth. Supporting the socio-ecological model (Sameroff, 2010), our findings captured five chronic stressors at multiple levels, including interpersonal (discrimination, foreigner stress), family (language brokering stress, economic stress), and neighborhood (neighborhood violence/non-safety) levels. These constructs were positively correlated with one another, supporting the theoretical and empirical evidence for the interrelatedness of transcultural and more culture-specific stressors (Kim et al., 2018). Moreover, it is notable that the latent constructs of such stressors were maintained across three waves, indicating the developmental stability of various types of stressors in Mexican-origin youths across waves.

Our second aim was to explore the associations between each stressor and internalizing symptoms among Mexican-origin youth from early adolescence to late adolescence/emerging adulthood. We found significant links between each type of stressor and adolescent anxiety and depressive symptoms, even after accounting for the influence of other stressors and covariates in our model. This suggests the unique contribution of these stressors to mental health outcomes at multiple socio-ecological levels.

At the interpersonal level, as expected, the positive association between racial/ethnic discrimination and internalizing symptoms was more pronounced during early and middle adolescence. One possible explanation is that adolescence represents a key developmental window in which youth become more attuned to social bias and power structures, while simultaneously engaging in deeper racial–ethnic identity formation (Umaña-Taylor et al., 2014) and developing critical consciousness related to race/ethnicity (Mathews et al., 2020). Empirical studies have found that perceived discriminate on among Mexican American youth increases during middle school (Hughes et al., 2006) and is associated with socioemotional adjustment (Benner & Kim, 2009).

On the other hand, the relationship between foreigner stress and internalizing symptoms was significant only during late adolescence and emerging adulthood. Consistent with our expectation, emerging adults who experienced higher foreigner stress exhibited higher levels of depressive and anxiety symptoms. This aligns with previous theory (Zou & Cheryan, 2017) and research (Armenta et al., 2013; Kim et al., 2011) indicating that greater foreigner stress is associated with more negative psychological outcomes. The notable association of foreigner stress with internalizing symptoms in emerging adulthood, but not earlier, contrasts with the pattern observed for discrimination. This may be due to the more subtle nature of foreigner stress compared to discrimination. While measures of discrimination focus on overt negative attitudes toward one’s racial–ethnic group, foreigner stress can be expressed inadvertently, even by individuals without harmful intentions (Armenta et al., 2013). Thus, Mexican-origin youth may experience foreigner stress more acutely in later developmental periods when they become more aware of subtle forms of foreigner objectification and implicit assumptions from others.

Unexpectedly, we found that in late adolescence and emerging adulthood, higher perceived discrimination was associated with *lower* internalizing symptoms, even after account for other stressors that were positively correlated with discrimination. Several explanations may account for this counterintuitive finding. First, young adults reporting higher discrimination may have developed stronger coping skills or resilience over time, buffering the emotional impact of discrimination (Benner et al., 2018). As youth gain autonomy, they also access broader social networks beyond school that provide support and opportunities to disclose and appraise discriminatory experiences, thereby mitigating distress (Dotterer et al., 2024; Ramos et al., 2022; Smart Richman & Leary, 2009). Second, young adults with fewer internalizing symptoms may be more attuned to recognizing discrimination rather than internalizing it. They may be more socially active, encounter diverse contexts, and attribute discrimination externally rather than to personal shortcomings. Third, this pattern may partly reflect a suppression effect, given that multiple stressors were controlled in the model. Alternatively, our findings could also reflect psychological reactivity (Runhardt, 2021): emerging adults exposed to chronic discrimination may downplay or normalize distress, either perceiving emotional expression as weakness or adapting to persistent adversity. Such responses represent not mere measurement error but potentially meaningful processes through which chronic discrimination shapes how distress is expressed and reported. Future research, particularly qualitative studies, could clarify how youth interpret survey items in these contexts, and whether they consciously minimize or reinterpret distress in light of discrimination.

At the family level, we observed pervasive effects of economic stress and language brokering stress on internalizing symptoms throughout adolescence and into emerging adulthood. This supports the family stress theory, indicating that family economic stress is a significant risk factor for children’s mental health across development (Conger & Conger, 2002). Additionally, the consistent negative impact of language brokering stress highlights its role as a culturally salient stressor, beyond general stressors like economic hardship (Kim, Schwartz, et al., 2018). The persistent effect of family-domain stressors suggests that the proximity and chronicity of stress exposure are crucial factors influencing adolescents’ mental health. Future studies should investigate how these factors interact with developmental timing to impact mental health.

At the neighborhood level, consistent with our hypothesis, the positive association between neighborhood violence/non-safety and internalizing symptoms was more pronounced during early adolescence, a period characterized by decreased reliance on parental protection and increased autonomy (Gee, 2021; Sameroff, 2010). Young adolescents who become more vigilant to neighborhood dangers may be more affected by unsafe environments. As they grow, emerging adults may develop coping strategies that reduce the impact of these stressors. Targeting neighborhood safety during early adolescence could be key to improving the emotional well-being of Mexican-origin adolescents (see next section for greater elaboration).

### Implications

We acknowledge that socio-ecological stressors are deeply rooted in structural inequities. Thus, effective interventions must extend beyond the individual or family level to address systemic conditions, while also considering developmental timing. As Kirkbride et al. (2024) note, systemic interventions are often the most effective for improving population-level mental health because they reduce exposure for large groups simultaneously, in contrast to individual interventions that reach fewer people. A comprehensive strategy should therefore combine structural reforms with developmentally tailored supports to buffer youth and families during sensitive periods of maturation.

At the structural and policy level, interventions should focus on creating safer and more equitable environments during developmental periods when youth are most susceptible to contextual stressors (Benoit et al., 2024). For example, improving neighborhood safety and reducing exposure to community violence during early adolescence, when youths’ sense of security and belonging are still forming, may yield long-term protective effects. Similarly, robust enforcement of anti-discrimination legislation and broader policies that reduce economic precarity among immigrant families (e.g., wage protections, expanded access to public benefits, targeted poverty reduction initiatives) are essential across development but may be particularly consequential during transition into adulthood, when economic stress and perception of foreignness often converge (Benoit et al., 2024). Importantly, efforts to improve neighborhood safety should prioritize community-driven approaches (e.g., investments in housing, infrastructure, and youth development programs; Kirkbride et al., 2024) rather than punitive strategies (e.g., over-policing) that may inadvertently reinforce discrimination or mistrust.

At the institutional level, interventions can be timed to coincide with key educational transitions. During early and middle adolescence – when discrimination appears most strongly linked to internalizing symptoms – schools can play a preventive role by adopting culturally responsive pedagogy, providing anti-bias training for teachers, and ensuring equitable disciplinary policies (Redmond et al., 2025). Establishing safe reporting mechanisms for discrimination and promoting programs that celebrate cultural diversity can further reduce bias and foster belonging during this critical period (Baysu et al., 2024; Eliot et al., 2010). Community-based organizations can also partner with families to offer language support services, thereby easing the burden on adolescents who serve as translators, and mitigating the stress associated with language brokering, which often peaks in early adolescence (Shen et al., 2022).

At the family and individual level, supports should likewise be developmentally informed (Crockett et al., 2025). In early adolescence, family-based programs that strengthen parent–child communication and emotional support can buffer the impact of discrimination and neighborhood stress. During middle adolescence, interventions that normalize help-seeking and teach adaptive coping may prevent the internalization of stress. As youth transition into adulthood, accessible, culturally sensitive and affordable mental health services (Crockett et al., 2025; Umaña-Taylor & Douglass, 2017) become increasingly important to support those already experiencing elevated anxiety or depression, particularly as new forms of foreigner stress and economic strain emerge.

Taken together, these implications suggest that interventions should be multi-level and developmentally timed, and structurally informed. Structural changes that address discrimination, economic stress, and neighborhood safety are essential for altering the root conditions that generate risk, while family- and individual-level supports can help youth and parents navigate stressors as they unfold across development. A multi-pronged approach is therefore most likely to yield durable improvements in the mental health of Mexican-origin adolescents from immigrant families.

## Limitations and future directions

Despite the strengths of this study, including comprehensive measures of stressors across socio-ecological levels and the use of longitudinal data, there are several limitations for future directions. First, the findings may not be generalizable to all Mexican-origin adolescents in the United States, as the sample was not nationally representative. Second, distinct from representativeness, our models estimate group-average, time-varying associations. Such averages can obscure meaningful person-specific heterogeneity and are not intended to characterize each adolescent. Likewise, factor structures derived at the sample level should not be interpreted as each individual’s psychometric organization (Molenaar & Campbell, 2009). Accordingly, our inferences target grouplevel developmental timing. Identifying individuallevel dynamics will require intensive longitudinal, personspecific designs (e.g., daily diary, ecological momentary assessment; personspecific modeling such as Ptechnique factor analysis or dynamic SEM), which were beyond the scope of the present study. Third, although our analyses leveraged longitudinal data across three waves, in this specification the TVEM approach estimates samplelevel, agevarying associations that primarily reflect betweenperson differences rather than withinperson change; as such, they do not establish temporal ordering or causality. TVEM is powerful for modeling developmental timing at the group level, but it does not separate within- from betweenperson effects or align with the different temporal scales at which stressors operate. For instance, structural and intergenerational stressors such as poverty or neighborhood safety often show little withinperson variance over typical study windows and are not well studied with short-term withinperson designs, whereas daily discrimination may fluctuate rapidly and is better captured with intensive repeatedmeasures designs. Our findings should thus be viewed as a grouplevel developmental map of when stressors are most strongly linked to internalizing symptoms, rather than as evidence of causal processes. Future research should build on this map using a plurality of methods: multilevel or random-intercept cross-lagged models to parse out both within- and between-person changes, intensive longitudinal and ecological momentary assessment designs to capture rapidly changing stressors, cohort and quasiexperimental designs (e.g., policy changes) to examine slowmoving structural factors, and qualitative or mixedmethods approaches to contextualize how stressors are experienced and interpreted. Such methodological pluralism is critical not only for advancing causal inference but also for ethical consideration when exposures cannot be manipulated, particularly for immigrantorigin families. Fourth, several stressor domains were assessed with a limited number of items due to the practical constraints of a longitudinal design, which required minimizing participant burden while ensuring broad coverage of socio-ecological stressors over time. For example, the measure of discrimination in academic settings demonstrated relatively low internal consistency, likely due to its brevity, as alpha reliability tends to be lower when fewer items are used. Similarly, domains such as skin color discrimination and economic strain were assessed with single items. While these indicators were incorporated into broader latent constructs using CFA to improve construct validity, single-item, and brief measures may still reduce the reliability and depth of assessment. Future studies should consider using validated multi-item scales or integrating qualitative methods to more comprehensively capture the complexity of these stress experiences.

## Conclusion

This study provides critical insights into the dynamic interplay between socio-ecological stressors and internalizing symptoms among Mexican-origin youth, highlighting how these associations shift across developmental periods. Importantly, our findings suggest that the sensitive period for interventions may vary by stressor type. For example, perceived discrimination and neighborhood unsafety were more impactful in early adolescence, while foreigner stress was linked to internalizing symptoms only in emerging adulthood. Economic stress and language brokering had pervasive effects throughout adolescence. These findings highlight the importance of early identification and targeted intervention based on the type and timing of stressors.

## Data Availability

The data that support the findings of this study are not publicly available but can be made available upon reasonable request to the corresponding author.

## Pre-registration

This study was not pre-registered due to its exploratory nature.
